# Identifying Indicators for the Early Detection of Internet Addiction Among University Students

**DOI:** 10.7759/cureus.84725

**Published:** 2025-05-24

**Authors:** Fujimaki Koichiro, Shigeru Morinobu

**Affiliations:** 1 Department of Occupational Therapy, Faculty of Health and Welfare, Prefectural University of Hiroshima, Mihara, JPN; 2 Department of Psychology, Faculty of Health and Wellness Sciences, Hiroshima International University, Hiroshima, JPN

**Keywords:** application use behavior, internet addiction, smartphones, social media usage situations, university students

## Abstract

Background: Internet use has been reported to cause disturbances in eating and sleeping habits. The results of a survey conducted by the Japanese Ministry of Education, Culture, Sports, Science and Technology indicated the need to investigate Internet addiction, which was defined as a dependence on the process of action, similar to gambling and shopping addictions. Therefore, this study aimed to identify the factors associated with a tendency to be dependent on the Internet and to examine the indicators for early detection of such dependence among university students.

Methodology: A questionnaire survey regarding the actual use of smartphones, the environment surrounding their use, and relationships with the tendency to depend on the Internet was conducted on 765 university students.

Results: Internet addiction test (IAT) scores were significantly correlated with the numbers of acquaintances communicated with on social media and the number of social media usage situations. The results of the multiple regression analysis showed that IAT scores could be predicted based on the number of social media use situations.

Conclusion: The findings of this study suggest that the greater the number of usage situations, the stronger the tendency to lose control of usage, and that the number of usage situations can be an indicator for identifying Internet addiction.

## Introduction

In recent years, the Internet has become an indispensable part of human life, and the number of users has been rapidly increasing. Students can use the Internet anytime and anywhere, owing to the wide adoption of campus-wide Internet access, smartphones, and data coverage.

Internet addiction has long been recognized as a behavioral problem that negatively affects daily life. Early studies, including those by Young [1], highlighted its similarities to substance-related and behavioral addictions such as gambling and compulsive shopping. Recent systematic reviews and meta-analyses have documented links between problematic Internet use and psychological distress, poor academic performance, interpersonal difficulties, and impaired work functioning [1]. These outcomes are often attributed to impaired self-control, withdrawal symptoms, and neglect of offline responsibilities, reflecting the disruptive impact of excessive Internet use on daily life. Furthermore, more recent research has provided a deeper understanding of its mechanisms and classification. For instance, the World Health Organization has officially recognized “Gaming Disorder” in the 11th revision of the International Classification of Diseases (ICD-11), emphasizing its global health relevance. In addition, several recent reviews have summarized the psychological, behavioral, and neurobiological correlates of problematic Internet use and smartphone addiction, providing updated frameworks for assessment and intervention [2,3].

Meanwhile, with the rapid spread of smartphones and mobile Internet access, the use of portable devices has significantly exceeded that of traditional personal computers (PCs). According to the Japanese Ministry of Internal Affairs and Communications, PCs were the primary devices used to access the Internet. However, with the rapid spread of smartphones and tablets, the smartphone penetration rate surpassed that of PCs, reaching 83.4% in 2019 [4]. According to more recent data from the same Ministry, the proportion of individuals using smartphones for Internet access in 2023 was approximately 95%, compared to 50% for PCs, indicating a continuing trend toward mobile-based Internet usage in Japan [5].

Early studies, such as those by Cheng et al. [6], highlighted significant differences in Internet usage between dependent and non-dependent users. However, with the proliferation of smartphones and mobile applications, recent research has shifted focus toward understanding the broader implications of excessive digital media use on mental health and daily functioning. For instance, Elhai et al. [7] emphasized the importance of social and tactile need fulfillment variables such as fear of missing out and need for touch as critical mechanisms that can explain problematic smartphone use and its association with depression and anxiety. Reflecting these concerns, Internet gaming disorder (IGD) was included in the fifth edition of the Diagnostic and Statistical Manual of Mental Disorders, Text Revision (DSM-5-TR) as a condition warranting further study, and the World Health Organization formally recognized gaming disorder in the ICD-11.

Previous studies have shown that university students spend over 25% of class time engaged with their smartphones for non-academic purposes. Furthermore, it was reported that phone usage was prevalent throughout the class, and phone distractions happened every three to four minutes for over a minute [8]. Several studies have reported that increased screen time is associated with decreased self-perceived attention [9]. Additionally, long-term measurement studies have demonstrated that frequent smartphone use during class interferes with academic tasks, which may negatively affect students’ well-being, productivity, and academic performance [8]. A large contributor to this effect is excessive social media use, which has been suggested to represent a vulnerability factor for problematic smartphone use [10]. Therefore, patterns of actual use - such as the amount of time spent online and the types of applications used - have been found to be weakly to moderately associated with Internet addiction in previous studies. These associations suggest that usage behavior may contribute to, but not fully explain, the risk of developing problematic Internet use.

The purposes of this study were to analyze university students’ actual use of the Internet - including smartphones, smartphone applications, and other devices - the environment surrounding their usage, and their relationships with Internet addiction. This study also aimed to clarify the factors associated with a strong tendency toward Internet dependence, in order to identify indicators for the early detection of Internet addiction.

## Materials and methods

Participants

This study employed a cross-sectional design and was conducted over four distinct periods: annually from April to July between 2015 and 2018. The target population consisted of first-year students enrolled in the Faculty of Health and Welfare at Hiroshima Prefectural University, Japan. Students surveyed between 2015 and 2018 belonged to different cohorts, and no students were surveyed in both periods.

A convenience sampling method was used. All students present in the relevant academic years were invited to participate voluntarily during regularly scheduled class hours. Questionnaires were distributed to 765 students, and valid responses were obtained from 738 students (response rate: 96.5%). After excluding incomplete responses, data from 690 students who fully completed the Internet Addiction Test (IAT) [6] were included in the final analysis.

The inclusion criteria were (1) current enrollment in the university during the survey period and (2) ability to provide informed consent.

The exclusion criteria were (1) incomplete responses to key items on the questionnaire, particularly the IAT, and (2) withdrawal of consent during the survey process.

This study was approved by the ethics committee of the Prefectural University of Hiroshima (approval number: 17MH012). Written informed consent was obtained from all participants prior to participation.

Survey form

Based on a previous survey conducted by the Ministry of Internal Affairs and Communications [11], we developed, distributed, and collected a five-item questionnaire on smartphone app use and Internet addiction tendencies among university students. There were five items on age at initial Internet use, time spent on the Internet by device, time spent by content used on Internet, number of people interacting with social media, and number of social media usage situations.

The number of social media usage situations was calculated by summing the number of different daily situations (e.g., while eating, while walking) in which the participant reported using social media. A total of 12 options were presented, including “(1) Immediately after waking in the morning” through “(11) While riding a bicycle” and “(12) None of the above.” The final score was derived by counting all selected situations, except option 12, resulting in a possible score range of 0-11. In cases where participants selected multiple overlapping situations (e.g., both “while watching television” and “while eating”), each selected option was counted as a separate usage situation regardless of whether the situations might occur simultaneously. This approach allowed for a standardized measure of the number of contexts in which social media was used throughout the day.

Young’s IAT

Internet addiction was assessed using the Japanese version of Young’s IAT [6], which was adapted by the Kurihama Medical and Addiction Center (Yokosuka, Japan). This version has been validated in Japanese populations and demonstrates high internal consistency, with a reported Cronbach’s alpha of approximately 0.91 [12]. The IAT consists of 20 items rated on a five-point Likert scale (from 1 (not at all) to 5 (always)), yielding a total score range of 20-100. On the basis of the total score obtained on the test, the individual is placed into one of two categories: normal Internet use (20-49 points, Category 1) and potential problematic Internet use (over 50 points, Category 2) [12]. No custom-developed instruments were used in this study.

Analytical methods

Spearman’s rank correlation coefficients were calculated between IAT scores and age of initiation by device, time spent on the Internet by device, time spent on the Internet by usage content, number of people interacting on social media, and number of social media use situations. Values of p < 0.05 were considered to indicate statistical significance. Since 24 variables were assessed, the statistical significance for multiple correlations between IAT score and the 24 variables was further evaluated using the Bonferroni p value (0.05/24 = 0.0021) to control for the family-wise error rate. Variable selection was conducted to select factors that predicted Internet dependence. Multiple linear regressions using the stepwise procedure were performed to determine predictors of IAT scores. All dimensions of the survey items were selected and represented as independent variables in the multiple linear regression analysis. A two-sided significance level of 0.01 was used for the multiple regression analysis.

We also calculated the percentage of respondents in each of the two categories according to their level of Internet addiction, as well as whether they used social media in each usage situation. Chi-square analysis was conducted for the bias in the frequency of responses for each situation. The significance level was set at p < 0.01. All statistical analyses were performed using PASW Statistics version 26.0 software (SPSS Japan, Tokyo, Japan).

## Results

The characteristics of the participants are shown in Table 1. Written informed consent was obtained from all participants prior to the study. Questionnaires were distributed to 765 students, and responses were obtained from 738 (96.5%). We analyzed the data from 690 individuals who completed all of the items on the IAT. In total, 535 (77.5%) students were classified as Category 1 and 155 (22.5%) as Category 2. The mean IAT score was 40.59 points (Table 2).

**Table 1:** Baseline characteristics of participants Data are presented as mean ± SD unless otherwise indicated. PC, Personal Computer

Characteristics	Mean values (standard deviation)
Participants, No.	690
Age (years)	19.82 ± 1.86
Years of education (years)	12.77 ± 0.56
Age at initial Internet use	10.00 ± 3.56
Time spent on the Internet by device (minutes per day)	
Personal Computer	35.46 ± 51.54
Smartphone	138.77 ± 102.97
Time spent on content used on the Internet (minutes per day)	
Browsing online video platforms on a PC	15.81 ±35.97
Browsing online video platforms on a smartphone	26.70 ± 42.66
Viewing social media on a PC	3.66 ± 13.67
Viewing social media on a smartphone	49.28 ± 57.04
Posting on social media on a PC	1.09 ± 9.63
Posting on social media on a smartphone	14.74 ± 30.45
Making free calls on social media on a PC	0.24 ± 2.92
Making free calls on social media on a smartphone	8.89 ± 27.29
Playing online games on a PC	3.31 ± 25.29
Playing online games on a smartphone	18.57 ± 33.45
Viewing a news site on a PC	2.82 ± 13.99
Viewing a news site on a smartphone	8.77 ± 12.78
Viewing a blog on a PC	0.54 ± 3.21
Viewing a blog on a smartphone	5.06 ± 19.72
Number of people interacting with social media	
Family	1.99 ± 1.47
Friends from the school are currently enrolled	8.54 ± 13.14
Friends from a previous school	5.58 ± 11.40
Friends made through activities outside of school (Part-time job, club activities, hobby activities, etc.)	3.35 ± 14.46
Acquaintances communicated with on social media and subsequently met in person	0.53 ± 3.02
Friends on social media only	1.97 ± 17.03
Number of social media usage situations (Alternatives: 1. Immediately after waking in the morning, 2. Before going to bed, 3. While watching television, 4. While eating, 5. While in class, 6. School recess, 7. While studying at home 8. While bathing, 9. In the toilet, 10. While walking, 11. While riding a bicycle, 12. Does not apply to 1-11)	5.31 ± 2.36

**Table 2:** Average score for the Internet Addiction Test score and each category Data are presented as mean ± SD unless otherwise indicated. IAT, Internet Addiction Test

IAT score	Mean values (standard deviation)
Overall average score	40.59 ± 12.76
Score for Category 1	35.21 ± 7.50
Score for Category 2	59.17 ± 9.20

The mean (± standard deviation) age of the participants was 19.82 ± 1.86 years. The mean age at initial Internet use was 10.00 ± 3.56 years (Table 1). A weak but statistically significant negative correlation was observed between IAT scores and age at initial Internet use (r = -0.145, p < 0.001), indicating that students who began using the Internet at a younger age tended to have slightly higher levels of Internet addiction (Table 3).

**Table 3:** Correlation between IAT score and survey items (correlation coefficients and p) The p-values are presented in their raw, uncorrected form, but the statistical significance for multiple comparisons was further corrected using the Bonferroni p value (0.05/24 = 0.0021) to control the family-wise error rate, and p < 0.0021 are deemed significant (*). IAT, Internet Addiction Test; PC, Personal Computer

Variables	IAT (score)	
	Correlation coefficient (r)	p-value
Age at initial internet use	-0.145	<0.001*
Time spent on the Internet by device (minutes per day)		
Personal computer	0.067	0.080
Smartphone	0.232	<0.001*
Time spent on content used on the Internet (minutes per day)		
Browsing online video platforms on a PC	0.086	0.025
Browsing online video platforms on a smartphone	0.194	<0.001*
Viewing social media on a PC	0.029	0.458
Viewing social media on a smartphone	0.148	<0.001*
Posting on social media on a PC	0.119	0.002
Posting on social media on a smartphone	0.133	0.001*
Making free calls on social media on a PC	-0.016	0.681
Making free calls on social media on a smartphone	0.086	0.024
Playing online games on a PC	0.093	0.016
Playing online games on a smartphone	0.125	0.001*
Viewing a news Site on a PC	0.003	0.929
Viewing a news Site on a smartphone	-0.004	0.927
Viewing a blog on a PC	0.074	0.056
Viewing a blog on a smartphone	0.049	0.202
Number of people interacting with social media		
Family	0.050	0.196
Friends from the school currently enrolled	-0.068	0.075
Friends from a previous school	-0.005	0.902
Friends made through activities outside of school (Part-time job, club activities, hobby activities, etc.)	0.064	0.095
Acquaintances communicated with on social media and subsequently met in person	0.216	<0.001*
Friends on social media only	0.224	<0.001*
Number of social media usage situations (Alternatives: 1. Immediately after waking in the morning, 2. Before going to bed, 3. While watching television, 4. While eating, 5. While in class, 6. School recess, 7. While studying at home, 8. While bathing, 9. In the toilet, 10. While walking, 11. While riding a bicycle, 12. Does not apply to 1-11)	0.368	<0.001*

The average times spent using a PC and smartphone were 35.46 ± 51.54 and 138.77 ± 102.97 minutes per day, respectively (Table 1). No correlations were found between IAT scores and time spent using a PC (Table 3). On the other hand, a significant correlation was observed between IAT scores and time spent using a smartphone (r = 0.232, p < 0.001).

Significant positive correlations were found between IAT scores and time spent browsing online video platforms (r = 0.194, p < 0.001), viewing social media (r = 0.148, p < 0.001), posting on social media (r = 0.133, p = 0.001), and playing online games on a smartphone (r = 0.125, p = 0.001). No significant correlations were observed between IAT scores and other content on the Internet (Table 3).

A significant positive correlation was found between IAT scores and the number of acquaintances communicated with on social media and subsequently met in person (r = 0.216, p < 0.001). In addition, a significant correlation was found between IAT scores and the number of friends on social media only (r = 0.224, p < 0.001). No significant correlations were observed between IAT scores and the number of other people interacting with them on social media (Table 4). A significant correlation was found between IAT scores and the number of social media usage situations (r = 0.368, p < 0.001).

**Table 4:** Multiple regression analysis for the IAT scores IAT, Internet Addiction Test, *Statistically significant

	Standardized coefficients β	F(df1, df2)	p	Adjusted R2
Number of social media usage situations (Alternatives: 1. Immediately after waking in the morning, 2. Before going to bed, 3. While watching television, 4. While eating, 5. While in class, 6. School recess, 7. While studying at home, 8. While bathing, 9. In the toilet, 10. While walking, 11. While riding a bicycle, 12. Does not apply to 1-11)	0.349*	F(1, 688) = 95.675	<0.001	0.121

The results of the multiple regression analysis for IAT scores are shown in Table 4. A stepwise multiple linear regression analysis was conducted with IAT scores as the dependent variable and several predictors as independent variables: age at initial Internet use, average daily smartphone use time, types of smartphone use (e.g., video viewing, social networking, gaming), number of social media acquaintances met in person or only online, and the number of social media usage situations. IAT scores were significantly predicted by the number of social media usage situations: F(1, 688) = 95.675, p < 0.001, Adjusted R² = 0.121. The results also revealed that media usage situations differed among IAT score categories. Table 5 shows that significantly more students in Category 2 (78.1%) reported Internet use immediately after waking in the morning compared with Category 1 (57.9%) (χ²(1) = 20.751, p < 0.001). Similarly, a significantly higher percentage of students in Category 2 reported Internet use while eating (χ²(1) = 24.118, p < 0.001), while in class (χ²(1) = 15.694, p < 0.001), and while studying at home compared with Category 1 (χ²(1) = 26.066, p < 0.001). Significantly higher percentages of students in Category 2 also reported Internet use while bathing (χ²(1) = 12.227, p < 0.001), while in the toilet (χ²(1) = 16.443, p < 0.001), and while walking compared with Category 1 (χ²(1) = 21483, p < 0.001). In addition, a significantly higher percentage of students in Category 2 (18.1%) reported Internet use while riding a bicycle compared with Category 1 (8.4%) (χ²(1) = 11.838, p = 0.001).

**Table 5:** Relationship between social media usage situations and categories of IAT scores Note: Values in the parentheses are percentages. IAT, Internet Addiction Test, *Statistically significant at the level of p < 0.01.

		Category 1 (n = 535)	Category 2 (n = 155)	χ²(df)	Adjusted p-value
Immediately after waking in the morning	No	225 (42.1)	34 (21.9)	χ²(1) = 20.751	< 0.001*
	Yes	310 (57.9)	121 (78.1)		
Before going to bed	No	51 (9.5)	13 (8.4)	χ²(1) = 0.187	0.665
	Yes	484 (90.5)	142 (91.6)		
While watching television	No	138 (25.8)	34 (21.9)	χ²(1) = 0.956	0.328
	Yes	397 (74.2)	121 (78.1)		
While eating	No	391 (73.1)	81 (52.3)	χ²(1) = 24.118	< 0.001*
	Yes	144 (26.9)	74 (47.7)		
While in class	No	307 (57.4)	61 (39.4)	χ²(1) = 15.694	< 0.001*
	Yes	228 (42.6)	94 (60.6)		
School recess	No	62 (11.6)	13 (8.4)	χ²(1) = 1.272	0.259
	Yes	473 (88.4)	142 (91.6)		
While studying at home	No	304 (56.8)	52 (33.5)	χ²(1) = 26.066	< 0.001*
	Yes	231 (43.2)	103 (66.5)		
While bathing	No	493 (92.1)	128 (82.6)	χ²(1) = 12.227	< 0.001*
	Yes	42 (7.9)	27 (17.4)		
In the toilet	No	409 (76.4)	93 (60.0)	χ²(1) = 16.443	< 0.001*
	Yes	126 (23.6)	62 (40.0)		
While walking	No	355 (66.4)	71 (45.8)	χ²(1) = 21.483	< 0.001*
	Yes	180 (33.6)	84 (54.2)		
While riding a bicycle	No	490 (91.6)	127 (81.9)	χ²(1) = 11.838	0.001*
	Yes	45 (8.4)	28 (18.1)		

## Discussion

Based on the results of the questionnaire, 77.5% of the participants were classified as Category 1 and 22.5% as Category 2. Category 1 represents individuals with normal or mild Internet use, while Category 2 includes individuals exhibiting a tendency toward problematic Internet use, as defined by the scoring criteria of the IAT. Of the 12,446 high school students recruited from four cities in Guangdong Province in China and invited to participate in the previous research, 87.8% of the participants were classified as Category 1 and 12.2% as Category 2, according to the IAT [13]. In comparison, our study conducted in Japan found a higher proportion of students in Category 2 (22.5%), suggesting potential cultural, educational, or technological differences influencing Internet usage behavior between the two countries. Compared with the results of that survey, the percentage of students at our university who tended to be dependent on the Internet was higher than that of the previous large-scale study.

A significant negative correlation was observed between IAT scores and age at initial Internet use, indicating that participants who began using the Internet at a younger age tended to have higher levels of Internet addiction. This finding is consistent with the results of Kamal et al. [14], who reported that students with problematic Internet use had an earlier age of initial Internet exposure compared to normal users. Although the exact reason for this is unclear, it may mean that exposing children to the Internet at a later age could be a protective factor against Internet addiction. The family environment is also important for protecting children from excessive Internet exposure. Parents should enforce measures regarding the Internet use of their children. In the present study, our results were consistent with those from previous studies in regard to a simple question asking about the age at initial Internet use. This finding is consistent with previous studies showing that earlier exposure to Internet use may increase the risk of problematic usage patterns later in life. Although the observed correlation was weak, it suggests that younger initiation may be a contributing factor to developing higher IAT scores, possibly due to prolonged habitual use or early reinforcement of online behaviors.

IAT scores were significantly correlated with time spent on the Internet on smartphones, but not PCs. The defining features of a smartphone, such as being portable, quick, convenient, and private, may facilitate access to certain problematic behaviors and the corresponding rewards received that make the behaviors more frequent [15].

In the present study, IAT scores were associated with time spent browsing online video platforms on a smartphone. The amount of smartphone use for seeking entertainment was positively related to a dependence on smartphones [16]. Regarding types of media content, the students who used smartphones for social media, games, and entertainment were more likely to be addicted, whereas those who used smartphones for study-related purposes were not [17]. Entertainment-related use had positive effects on Internet addiction; this is an interesting finding given that music and video use, compared with other smartphone functions, is closest to the function of traditional entertainment media (e.g., television and radio). Furthermore, past communication research has noted that television watching can be addictive [17].

Significant positive correlations were observed between IAT scores and time spent viewing and posting on social media on a smartphone. As early adulthood is a critical developmental stage marked by increasing independence and identity formation, university students may be particularly susceptible to developing addiction-like symptoms through daily social media use. For instance, Peris et al. [18] considered the following four types of risk behavior regarding social networking and Internet addiction in adolescents on a subclinical level: Internet addiction symptoms, social media use, geek behavior, and nomophobia, which refers to the discomfort or anxiety caused by the non-availability of a mobile phone, PC, or any another virtual communication device. Messaging apps have increased the prevalence of nomophobia, especially among teenagers, the age group most affected by this problem. Users addicted to smartphones and social media experience higher levels of cognitive absorption, particularly females, when using social media. Furthermore, time spent viewing and posting on social media on a smartphone appeared to be related to both a fear of missing out, which is the fear of being excluded from rewarding social experiences, and nomophobia; these have both been evidenced in the smartphone literature as reinforcing use and triggering a need to be in constant contact [19]. There may be a situation where nomophobia intervenes and forces such users to check social media on their smartphones.

A significant positive correlation was observed between IAT scores and time spent playing online games on a smartphone. The risk factors for games were money spent on gaming and weekday game time. Users with IGD spend more time playing Internet games than do normal gamers [20]. The Internet can be used for many purposes, but an increasing number of studies have demonstrated that gaming is a distinct form of excessive Internet use and is associated with unique harms [21].

A significant positive correlation was found between IAT scores and the number of acquaintances communicated with on social media and subsequently met in person (r = 0.216, p < 0.001). This suggests that students with higher levels of Internet addiction (as measured by IAT scores) tend to have more acquaintances met through social media, potentially reflecting a greater tendency to engage in online social interactions rather than face-to-face communication. Additionally, correlations were observed between IAT scores and smartphone use (r = 0.232, p < 0.001) but not with PC use (r = 0.067, p = 0.080). These findings indicate that smartphone use, particularly for social media and entertainment, is more strongly associated with higher IAT scores compared to traditional PC usage. University students with IGD have been reported to experience lower levels of self-perceived social support, greater reliance on online-only friendships, and reduced health-related quality of life compared to their peers without IGD. These findings suggest that problematic gaming behaviors during early adulthood may have significant impacts on both psychological well-being and social functioning [22]. A positive association was found between problematic cell phone use and having a larger circle of friends, which may reflect a greater tendency toward extroversion among problematic cell phone users. Sociability is one of the major defining features of extroversion, and extroverts consequently tend to have larger circles of friends and social networks, which might promote a greater use of mobile phones, since these devices appear to serve as a tool of social influence [23]. A previous study suggested that a low quality of interpersonal relationships can expose adolescents to an increased risk of developing problematic Internet use [24]. The Internet provides a space for users to escape from reality and seek acceptance [25]. The fact that IAT scores were correlated with the number of friends met on social media only suggests that students with Internet addiction are more likely to avoid real-life contact. In addition, our findings suggest that social media is becoming one of the most important means of social interaction for students.

IAT scores were significantly predicted by the number of social media usage situations (Table 4). Students use mobile phones while walking to class, riding on a bus, or waiting for an elevator. These “micro time slots” in which people can engage in a vast array of online activities were not previously available [26]. A previous study reported that 96.8% of students used smartphones during lectures, classes, and meetings. According to the students, the advantages of using a smartphone included its mobility, easy access, ease of use, and real-time access to information [27].

Significantly higher percentages of students in Category 2 reported Internet use while bathing, while in the toilet, and while walking compared with Category 1 (Table 5). A previous study also reported that proneness to smartphone addiction was positively correlated with a declared frequency of smartphone use while walking [28]. The portability of mobile touch screen devices (MTSDs) has enabled adolescents to use them ubiquitously; almost everywhere and anywhere is possible, even in various areas of the house, including the toilet, as reported by adolescents [29]. The use of social media while bathing, while in the toilet, and while walking could be limited to smartphones, not to PCs. Hence, the high usage rate among students in Category 2 may have been due to the effects of portability on Internet addiction.

Significantly higher percentages of students in Category 2 reported Internet use immediately after waking in the morning (78.1%) compared with Category 1 (57.9%). To many of the adolescents, MTSDs seemed irresistible; they often felt inclined to use MTSDs, especially when such devices were within their sight and reach [29]. Thus, there appears to be a need to address the irresistibility of MTSDs reported by students, which causes them to use their smartphones immediately after waking in the morning.

A significantly higher percentage of students in Category 2 reported Internet use while in class, while riding a bicycle, and while studying at home, compared with Category 1. Usage while in class induces negative outcomes such as reduced academic performance [30], which are associated with multitasking during MTSD use. The risks of mobile phone use while cycling are clear. The effects of cognitive demand on speed and peripheral vision have been reported [31]. Using a smartphone despite knowing the above risks suggests a lack of self-control, which may account for high and frequent use. A significantly higher percentage of students in Category 2 reported Internet use while eating compared with Category 1. The amount of time spent online has been reported to be related to changes in sleeping and eating patterns [32]. The relationship between dependence on eating and Internet use at the same time has been reported. The habit of snacking while using the Internet has also been reported to be associated with Internet addiction [33]. Thus, Internet addiction also affects the dietary behaviors and lifestyle characteristics of university students.

It is important to raise students’ awareness of the problems associated with using the Internet in an increasing number of daily situations. As students begin to engage with the Internet during various activities - such as while studying, eating, walking, or socializing - it may become more difficult to regulate their usage, leading to habitual multitasking, reduced concentration, sleep disturbances, and decreased academic performance. Therefore, strategies that help students use smartphones or PCs in a more intentional and limited manner, without placing them at risk of compulsive or addictive behavior, need to be developed.

This study had some limitations. First, it was conducted at a single university and included only students from the Faculty of Health and Welfare, which may limit the generalizability of the findings. Additionally, although the sample size was relatively large (N = 690), the study was cross-sectional in nature and relied solely on self-reported data, which may introduce reporting bias or inaccuracies in recall. Furthermore, detailed demographic information - such as marital status, living arrangements, and socioeconomic background - was not collected. Future studies should consider including these variables to allow for a more comprehensive analysis of contextual and lifestyle factors associated with Internet addiction.

Additionally, cultural norms, educational environments, and access to digital technology may influence the manifestation and perception of Internet addiction. As this study was conducted among Japanese university students, the findings should be interpreted within this specific cultural and institutional context. Future studies are encouraged to conduct cross-cultural comparisons to examine whether the identified patterns and indicators are generalizable to different populations and settings.

We acknowledge that non-response bias is a potential limitation of this study. Although the overall response rate was high, students who were absent during the data collection sessions were not included. This may have introduced sampling bias, as their Internet usage behaviors could differ from those who were present. We have noted this as a limitation and recommend that future studies consider strategies to include absent students or assess the potential impact of non-response.

We also acknowledge the conceptual distinction between smartphone addiction and Internet addiction, despite their considerable overlap. In this study, we used the IAT as a broad measure of online dependency. However, the IAT does not specifically differentiate between types of device usage. We recognize this as a limitation and suggest that future research consider employing assessment tools specifically designed to measure smartphone addiction in order to more accurately capture the nuances of device-specific dependency.

Despite these limitations, the study also had several strengths. It used a validated and widely adopted measure of Internet addiction (the IAT) and included detailed behavioral and contextual variables, such as the number of social media usage situations and average screen time. The relatively high response rate (96.5%) also supports the reliability of the dataset.

Future research should consider longitudinal designs to examine causal relationships and explore additional psychosocial factors such as self-regulation, mental health status, and academic outcomes. Moreover, expanding the population to include students from multiple universities or different academic disciplines could enhance the generalizability of the findings.

## Conclusions

Smartphone use is integrated into the daily routines of university students. Students frequently check their smartphones and browse social media in various situations. Checking social media during class and while walking or riding a bicycle can cause academic problems and pose a danger to oneself and others, respectively. The fact that people cannot stop checking social media using smartphones and other devices, even though they are aware of such problems, suggests problematic Internet use. The results of this study suggest that limiting smartphone use to some extent in regard to browsing social media is an effective strategy to prevent the progression to addiction.
